# CAP modifies the structure of a model protein from thermophilic bacteria: mechanisms of CAP-mediated inactivation

**DOI:** 10.1038/s41598-018-28600-w

**Published:** 2018-07-05

**Authors:** Pankaj Attri, Jeongmin Han, Sooho Choi, Eun Ha Choi, Annemie Bogaerts, Weontae Lee

**Affiliations:** 1 Research Group PLASMANT, Department of Chemistry, University of Antwerp, Universiteitsplein 1, B-2610 Antwerp, Belgium; 2 Department of Biochemistry, College of Life Science & Biotechnology, Yonsei University, 134 Shinchon-Dong, Seodaemoon-Gu, Seoul, 120-749 Korea; 3 Department of Electrical and Biological Physics, Kwangwoon University, Seoul, 01897 Korea

## Abstract

Cold atmospheric plasma (CAP) has great potential for sterilization in the food industry, by deactivation of thermophilic bacteria, but the underlying mechanisms are largely unknown. Therefore, we investigate here whether CAP is able to denature/modify protein from thermophilic bacteria. We focus on MTH1880 (MTH) from *Methanobacterium thermoautotrophicum* as model protein, which we treated with dielectric barrier discharge (DBD) plasma operating in air for 10, 15 and 20 mins. We analysed the structural changes of MTH using circular dichroism, fluorescence and NMR spectroscopy, as well as the thermal and chemical denaturation, upon CAP treatment. Additionally, we performed molecular dynamics (MD) simulations to determine the stability, flexibility and solvent accessible surface area (SASA) of both the native and oxidised protein.

## Introduction

Cold atmospheric plasma (CAP) produces a cocktail of reactive species, including radicals, excited species, positive and negative ions, atoms, molecules, etc. that play a vital role in its anticancer and antibacterial activity^1–15^. CAP is also increasingly used in the field of wound healing, agriculture, water purification, etc.^16–23^. Various aspects contribute to the microbial inactivation capacity of CAP, such as UV photons produced in the plasma, the electrical field, reactive oxygen and nitrogen species (RONS), etc.^1,13–15^. In the gas phase, the RONS, UV photons and electric field create strong oxidative stress that deactivates the microbes through lipid peroxidation, enzyme inactivation, and DNA cleavage^15^. However, fluid mediated plasma treatment for inactivation of microbes is mainly influenced by the RONS generated in the gas phase or at the gas-liquid interface, that diffuse inside the liquid to induce the antimicrobial effects^15^. During plasma treatment, acidification of the plasma treated liquid is one of the critical factors responsible for the antimicrobial effect, but it is reported that acidic pH is not the main reason for the antimicrobial effect after plasma treatment^24,25^. The production of reactive species, such as peroxynitrite, during plasma treatment may be the main factor for the bactericidal effect, because the intracellular damage caused by peroxynitrite cannot be repaired by cells^26,27^.

CAP has been used among others to treat biofilms and planktonic bacteria^28–30^. In 2006, the first clinical plasma application was reported to treat diverse skin and soft tissue infections^20^. Since then, CAP is used to treat chronic wounds that exhibit nosocomial multidrug resistance, such as *vancomycin-resistant enterococci* (*VRE*), *methicillin-resistant Staphylococcus aureus* (*MRSA*), *Escherichia coli*, *Pseudomonas aeruginosa*, *Klebsiella pneumoniae*, *Acinetobacter baumannii*, *Clostridium difficile*, etc.^31–34^. In our earlier work, we have already shown that dielectric barrier discharge (DBD) and nanosecond pulsed plasma can kill the multidrug resistant *Staphylococcus aureus* (*S*. *aureus*) bacteria (Penicillum-resistant, Methicillin-resistant and Gentamicin-resistant), as well as wild type *S*. *aureus*^35^. However, the mechanisms that underpin the CAP action against the microorganisms are not yet fully understood. A few studies have investigated the effect of CAP on protein modification/degradation, such as heme degradation of horseradish peroxidase^36^, inactivation of lysozyme solution^37,38^, structural modification of myoglobin with or without co-solvents^39–41^, activation of lipase solution^42^, structural modification of hemoglobin with or without co-solvents^39^, inactivation of polyphenoloxidase (PPO) and peroxidase (POD)^43^, structural changes of α-chymotrypsin with or without co-solvent^44^, and inactivation of lactate dehydrogenase (LDH) enzyme^45^. These studies help in understanding the change in structure and function of microorganisms after CAP treatment.

Surprisingly, the effects of CAP on proteins from thermophilic bacteria, which denature at much higher temperature than regular mesophilic proteins, have not been closely examined. The thermophilic bacteria, which are a common problem in the manufacturing of milk powder and more in general in the food industry^46,47^. *Geobacillus spp*. is a thermophilic bacterium that forms biofilms, and when spores are present in raw milk, they survive during pasteurization and adhere to stainless steel surfaces. When the biofilm matures, the bacterial spores penetrate and contaminate the milk powder, which lowers the product value^48,49^. Likewise, spores of the thermophilic *Bacillus stearothermophilus* can survive during thermal processing towards a commercially sterile product (at a typical temperature of 121 °C) and spoil low acid canned foods, such as canned vegetable or fruit products^50^. The adaptation of thermophilic bacteria at high temperature is a combination of various factors, including functional acclimatization and genetic selection. It was demonstrated through proteomics that most of the thermophilic proteins contain a gene that encodes proteins with high thermostability. These thermostable proteins play a pivotal role in the glycolysis pathway, antitoxins, antioxidants, etc. Additionally, protein-protein interactions are also involved in thermal tolerance^51^. Moreover, proteins from thermophilic bacteria are known to be resistant to chemical denaturants^52^. Therefore, it is of great interest to investigate the capabilities of CAP for this purpose, as this can reveal whether CAP can deactivate thermophilic bacteria. To our knowledge, the latter has not been studied yet, but if it is possible, it would be very promising for applications in the food industry. CAP is already used for sterilization purposes in the food industry, but it is not clear whether it can also deactivate thermophilic bacteria. Hence, it is interesting to investigate the underlying mechanisms, to reveal the potential action of CAP on proteins from thermophilic bacteria.

The purpose of this study is thus to determine the effect of CAP on the structure of protein from thermophilic bacteria. The solution structures of thermophilic proteins are derived from *M*. *thermoautotrophicum* through genomics projects^53–57^. Among them, MTH1880 is a protein from thermophilic bacteria *M*. *thermoautotrophicum*, which grows at an optimum temperature of 65–70 °C. MTH1880 is a relatively small protein, it folds compactly at room temperature and behaves as a model for thermophilic proteins based on previous studies^56,58^. Additionally, the MTH1880 structure is widely studied and it is extremely stable in denaturing conditions^56,58^. Therefore, in this work, we used MTH1880 (or simply called MTH) as a model protein from thermophilic bacteria. We exposed MTH to dielectric barrier discharge (DBD) plasma for 10, 15 and 20 min in buffer solution. We carried out structural analysis of MTH before and after CAP treatment using circular dichroism (CD), fluorescence and ^1^H-^15^N NMR spectroscopy. Furthermore, we used CD spectroscopy to determine the change in chemical and thermal denaturation of MTH upon CAP treatment. In support of the experiments, we performed molecular dynamics (MD) simulations to compare the root-mean-square deviation (RMSD), root mean square fluctuation (RMSF), essential dynamics (ED) and solvent accessible surface area (SASA) of oxidised MTH (obtained upon plasma treatment) vs. native MTH, in order to evaluate the change in stability and flexibility of the protein.

## Results and Discussion

### Change in pH, temperature and reactive species after plasma treatment

**Figure 1:**
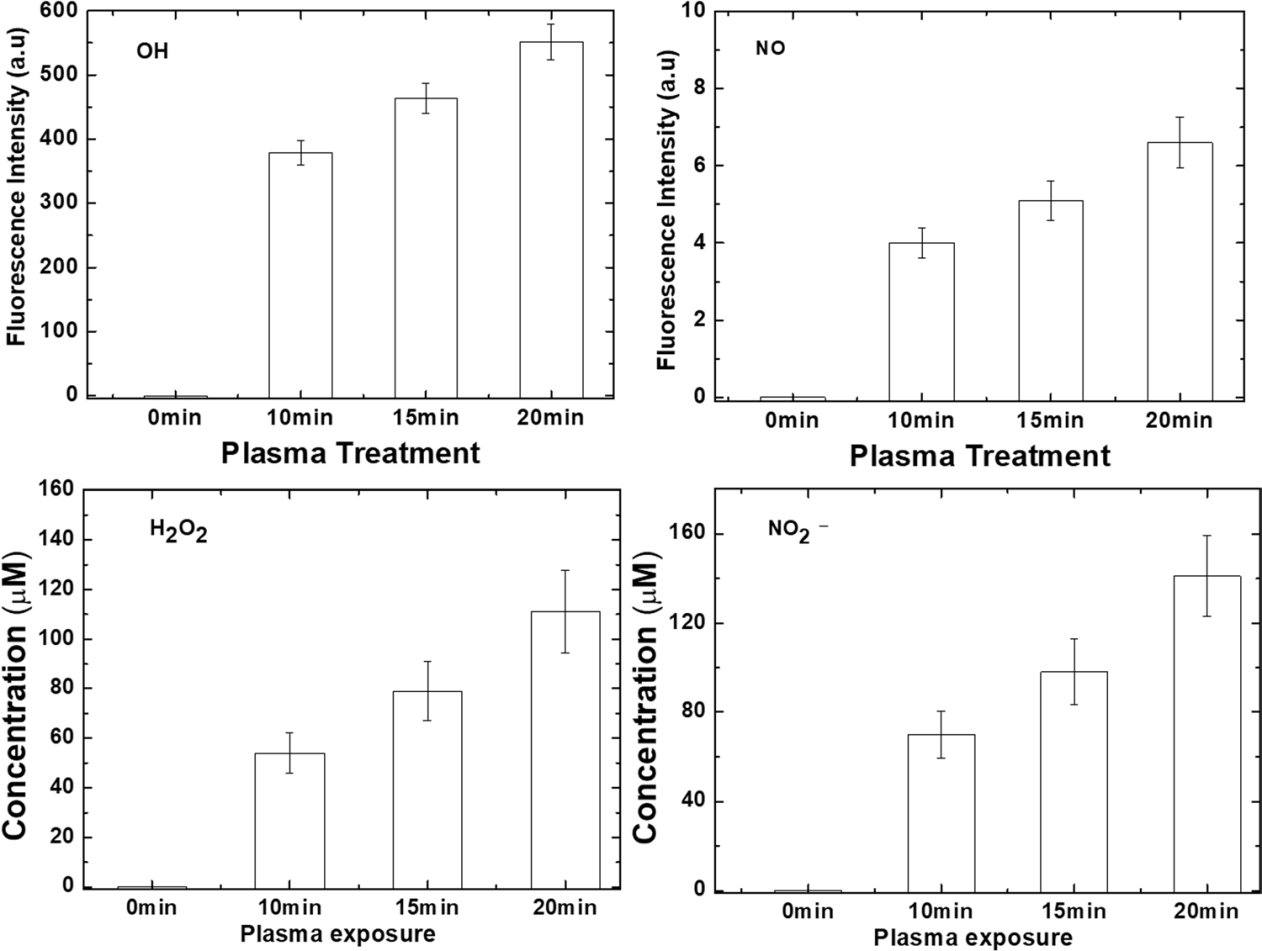
Fluorescence intensities or concentrations of different RONS generated during DBD treatment for 0, 10, 15 and 20 mins.

During plasma treatment, no change in pH and temperature of the protein solution was observed, as shown in Fig. S1. However, the concentration of reactive species changed with variation in treatment time. We measured the ^•^OH, and ^•^NO radicals, the H_2_O_2_ molecules and NO_2_^−^ ions after 10, 15 and 20 min of DBD plasma with air as feeding gas, as shown in Fig. 1. To determine the OH radical formation, we used the TA (terephthalic acid) analysis method^59^. The fluorescence intensity (due to formation of HTA that correlates with the OH concentration) increases with increasing plasma treatment time. A similar result is obtained for the fluorescence intensity of the NO radicals. Likewise, the concentrations of H_2_O_2_ and NO_2_^−^ in buffer exhibit the same behaviour. Thus, the production of these four RONS increases upon increasing CAP treatment time.

### CD and fluorescence analysis of MTH before and after plasma treatment

The MTH structure consists of antiparallel β-sheets and α-helix^58^. The region between β-sheets (β3 and β4) and helix 2 forms the hydrophobic core of MTH. Helix 1 is part of the hydrophobic core, while helix 2 at the C terminus packs against the β-sheets to cover the hydrophobic core residues. Loops B and D and loops C and E generate the highly acidic and basic surface, respectively^58^.

**Figure 2:**
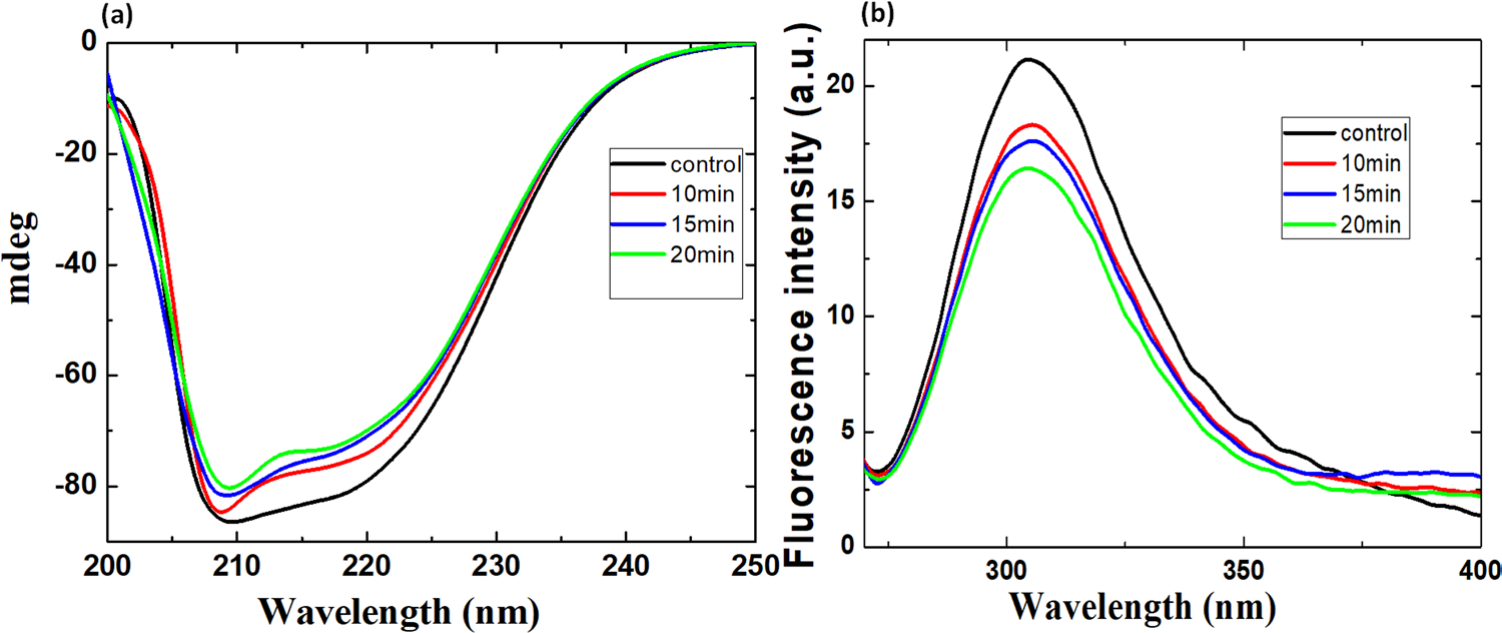
(**a**) Far CD spectra and (**b**) fluorescence spectra of MTH before (“control”) and after plasma treatment for different times.

The CD spectra of MTH without plasma treatment (“control”) revealed that the α-helix and β-sheet occupy 24% and 14% of the structure, respectively, as shown in Fig. 2a. After plasma treatment for 10 min, the volume occupied by the α-helix decreases to 20%, while that of the β-sheet increases to 19%. Plasma treatment of 15 min revealed no change in fraction of α-helix compared with 10 min plasma treatment, whereas the fraction of β-sheet slightly increased to 20%. After plasma treatment for 20 min, the volume occupied by the α-helix decreased to 19%, while that of the β-sheet increased to 23%. Thus, the fractions of α-helix and β-sheet slightly decrease and increase after plasma treatment, similar to previously reported work by our and other groups for various proteins^36,39^.

The intrinsic fluorescence spectrum of MTH before and after plasma treatment is shown in Fig. 2b. MTH contains two tyrosine residues (Tyr25 and Tyr59) with no Try residue. The intrinsic fluorescence in MTH is due to tyrosine. It reveals a high intrinsic anisotropy and is optimal to depict nanosecond motions in peptides and proteins using fluorescence lifetime^60^. However, it has a low fluorescence quantum yield, low extinction coefficient and low sensitivity upon changes in the surrounding environment^61^. Nevertheless, it is useful to investigate the structural and dynamic changes in MTH protein. Before plasma treatment, MTH exhibits a maximum emission spectrum at ≈304 nm, but the intensity drops gradually after plasma treatment for 10, 15 and 20 mins, as illustrated in Fig. 2b, indicating a slight change in surrounding environment. However, no significant shift in the fluorescence peak was observed, which can be attributed to the low sensitivity of tyrosine towards changes in the surrounding environment, as mentioned above.

Based on the CD and fluorescence analysis, we can conclude that the structure of MTH is modified or denatured. The drop in fraction of α-helix and the rise in fraction of β-sheet after plasma treatment can be attributed due to the modification of amino acids, which changes the conformation of the MTH protein. At the same time, the quenching of the fluorescence indicates a change in the environment near the Tyr residue or can be due to modification of Tyr. Thus, after plasma treatment the amino acids are possibly modified/oxidised, which decreases the interaction between them, resulting in opening of the protein structure.

### Influence of plasma on thermal and chemical stability of MTH

**Figure 3:**
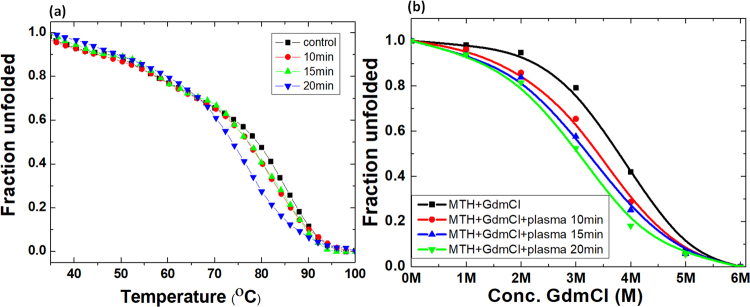
(**a**) Thermal denaturation and (**b**) chemical denaturation of MTH before (“control”) and after plasma treatment for different times.

Proteins exhibit a well-defined three-dimensional structure, characteristic for specific environmental conditions, while outside these environmental conditions, proteins display an unfolded state. We used CD spectroscopy to determine the change in melting temperature (T_m_) of MTH upon plasma treatment, to investigate the effect of plasma on the thermal stability of MTH. The melting temperature was analysed at 220 nm, before and after plasma treatment, as shown in Fig. 3a. Before plasma treatment, T_m_ was 79.2 °C, while after plasma treatment for 10, 15 and 20 mins, T_m_ changes to 78, 78.2 and 74 °C, respectively. Hence, no significant change in melting temperature of MTH was observed after plasma treatment for 10 and 15 mins, whereas after 20 min, T_m_ has dropped by 5 °C. Hence, plasma treatment can influence the thermal stability of protein from thermophilic bacteria, but only at higher treatment doses.

We also investigated the stability of protein against chemical denaturation before and after plasma treatment, as displayed in Fig. 3b, using GdmCl as denaturant. It is a well-known denaturant for the complete unfolding of protein, with stronger effect than urea^62^. We acquired far-UV CD spectra for MTH for 6 different GdmCl concentrations ranging from 1 to 6 M. For the native protein, the fraction unfolded was approximately unaffected up to 2 M GdmCl concentration, while complete unfolding was observed for 6 M GdmCl. The fraction of unfolding for the native protein was observed at ≈3.7 M GdmCl. After plasma treatment for 10, 15 and 20 min, the fraction of chemical unfolding occurred at ≈3.4, 3.2 and 3 M GdmCl. Thus, plasma seems to have some effect on the chemical denaturation of MTH, although the effect is quite limited.

### NMR analysis of MTH before and after plasma treatment

To better understand which amino acids are affected by plasma treatment, we applied ^1^H-^15^N NMR spectroscopy. In our previous work, we have shown that hydrophobic and electrostatic interactions play a key role in stabilizing the MTH structure^56^. The hydrophobic core is constituted by the Leu15/Pro16/Pro21/Val23/Leu40, Phe10/Leu15/Val23/Ile40/Ile42, Val23/Tyr25/Ile40/Ile42/Val53, Leu54/Leu69/Ala72/Leu84/Leu88, Val53/Val23/Ile40/Ile42, Leu56/Ala41/Tyr59/Leu69 and Leu84/Leu54/Leu81/Ile83 residues. Moreover, the residues Asp63-Arg66, Arg66-Glu70, Lys7-Glu70, Lys13-Asp36, Lys67-Glu71, Glu68-His87, Lys13- Asp38, and Glu71-Lys75 contribute to the salt bridges that stabilise the protein structure. CD and fluorescence analysis revealed some structural changes after 20 min DBD plasma treatment. Hence, we prepared the ^1^H-^15^N NMR samples as described in the Material and Method section, and treated the ^15^N-label sample with plasma for 20 min.

**Figure 4:**
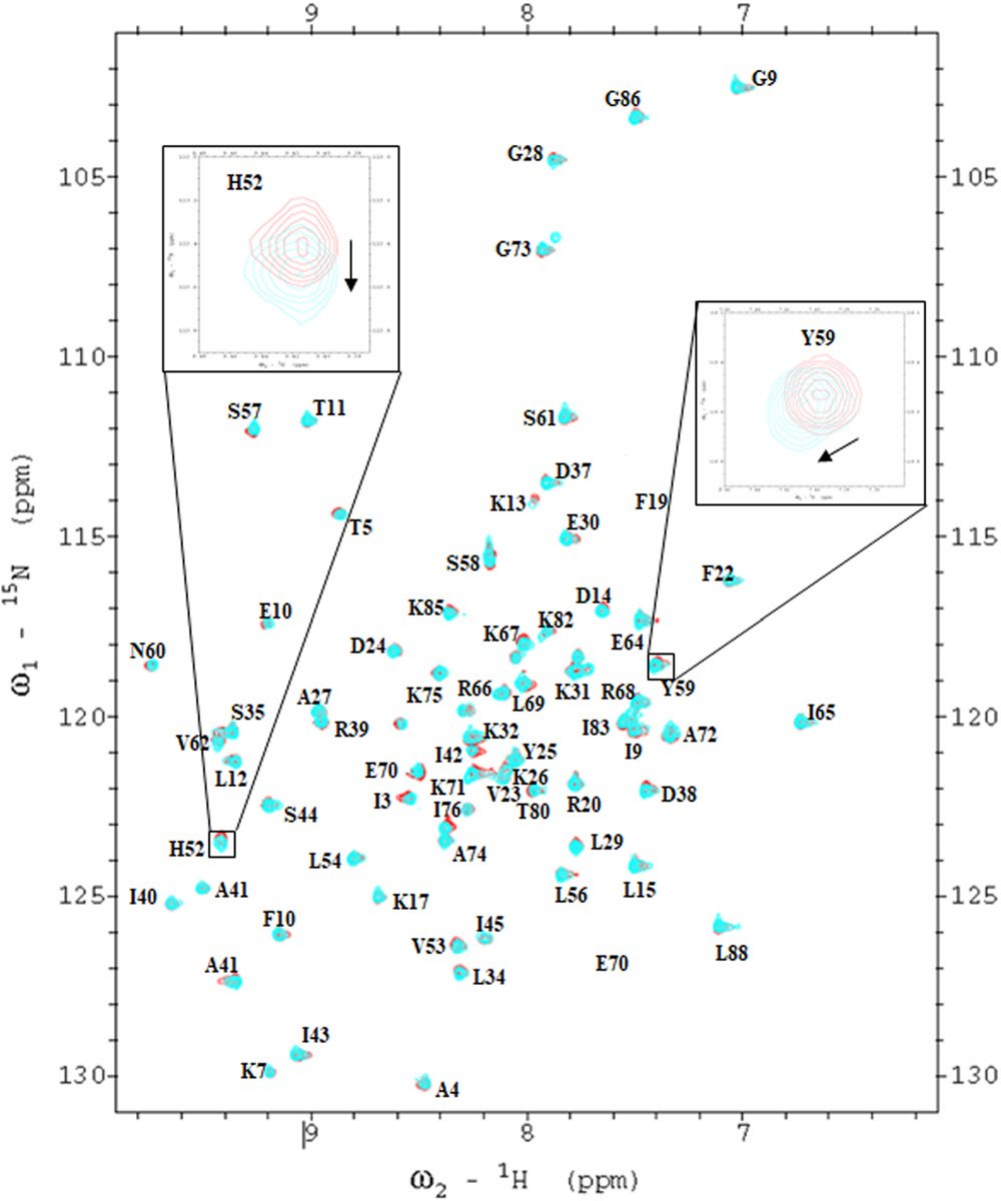
^1^H-^15^N NMR spectra of MTH before (red) and after 20 min plasma treatment (cyan). The major differences are enlarged in the insets.

The NMR spectra showed that after plasma treatment the major MTH amino acid peaks overlap with the control MTH amino acid peaks, as illustrated in Fig. 4. Only a few amino acid peaks show a shift in NMR after plasma treatment, such as His52 and Tyr59. If we compare the control and treated MTH NMR peaks, we see that the His52 peak shifts by 0.02 ppm and Tyr59 shifts by 0.03 ppm. The other amino acids did not show a significant peak shift in the ^1^H-^15^N NMR spectra. The Tyr59 peak shift can be correlated to the drop in fluorescence intensity, and is attributed to the oxidation of tyrosine. The His52 and Tyr59 peak shifts indicate a modification of the MTH structure, as also shown in the CD spectra. Most probably the oxidation of His52 and Tyr59 is due to reactions between His and singlet oxygen (^1^O_2_), and between Tyr and hydroxyl radicals (OH), respectively. Indeed, singlet oxygen and hydroxyl radicals are the key radicals for the bactericidal effect, as stated by Wu *et al*.^63^. Note that other amino acid peaks are also slightly shifted, but we have only considered the significant peak shifts of the amino acids.

### Molecular dynamics simulation of native and oxidised MTH

Based on the change in the NMR spectrum and previously reported oxidation of amino acids by CAP^64^ we performed MD simulations to calculate the RMSD, RMSF, ED and SASA of MTH before and after plasma oxidation, to evaluate the effect of plasma treatment on the stability and flexibility of the protein. The above ^1^H-^15^N NMR analysis shows a change in His52 and Tyr59. Therefore, we have modified these amino acids, as described in the Material and Method section, and we analysed the RMSD, RMSF, ED and SASA.

**Figure 5:**
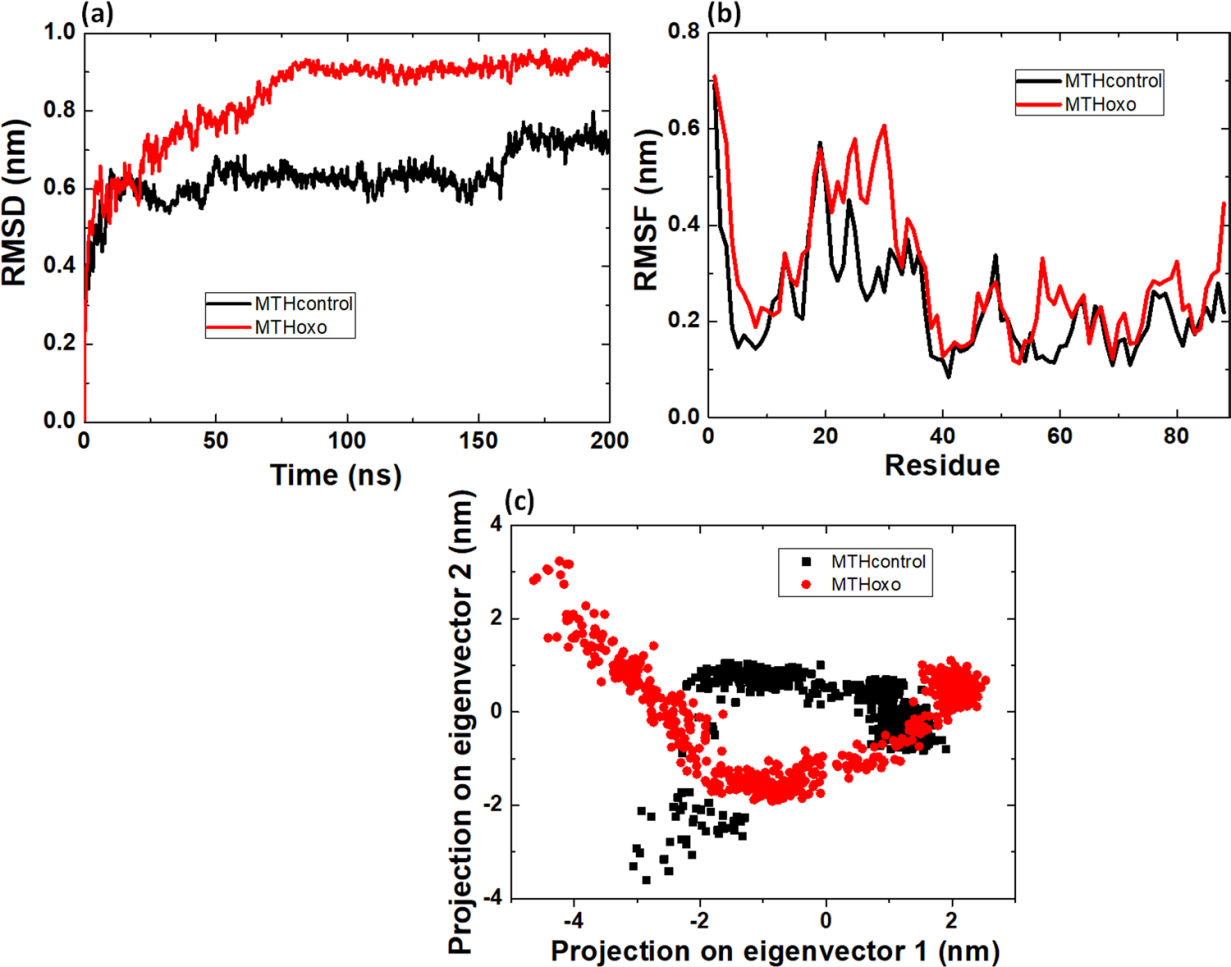
(**a**) RMSD, (**b**) RMSF and (**c**) projection of the motion in phase space along the first two principal eigenvectors values, of MTH control and MTHoxo.

Figure 5a shows the RMSD during 200 ns for native MTH (i.e., MTH control) and oxidized MTH (MTHoxo). The RMSD provides information on the degree of similarity between both protein structures. The average RMSD value for MTH control is 0.63 ± 0.06 nm, while it 0.83 ± 0.12 nm for MTHoxo. Figure 5b illustrates the RMSF, which describes the mean fluctuation per residue between two optimally-aligned structures, so it gives information on the average atomic mobility per residue of MTH. The average RMSF for MTH control and MTHoxo is 0.23 ± 0.09 and 0.29 ± 0.13 nm, respectively. Hence, both RMSD and RMSF values are higher for MTHoxo, which indicates that oxidation of MTH results in losing its stability, which also affects the structural orientation. In other words, after oxidation of the MTH protein, its degree of flexibility increases.

We also performed essential dynamics (ED) analysis to obtain a better view on the dynamical mechanical property of the MTH and MTHoxo structures. For ED analysis, we applied a simple linear transformation in the Cartesian coordinate space and the diagonalization of the covariance matrix yields a set of eigenvectors. These eigenvectors provide a vectorial illustration of every single component motion. The projection of trajectories obtained at 26 °C shows the motion of the native and oxidised MTH protein; see Fig. 5c. The overall flexibility of MTH control and MTHoxo was calculated by the trace of the diagonalized covariance matrix of the C_α_-atomic positional fluctuations, and was 4.07 and 7.37 nm^2^, respectively. Hence, the MTHoxo flexibility increased as compared to MTH control.

**Figure 6:**
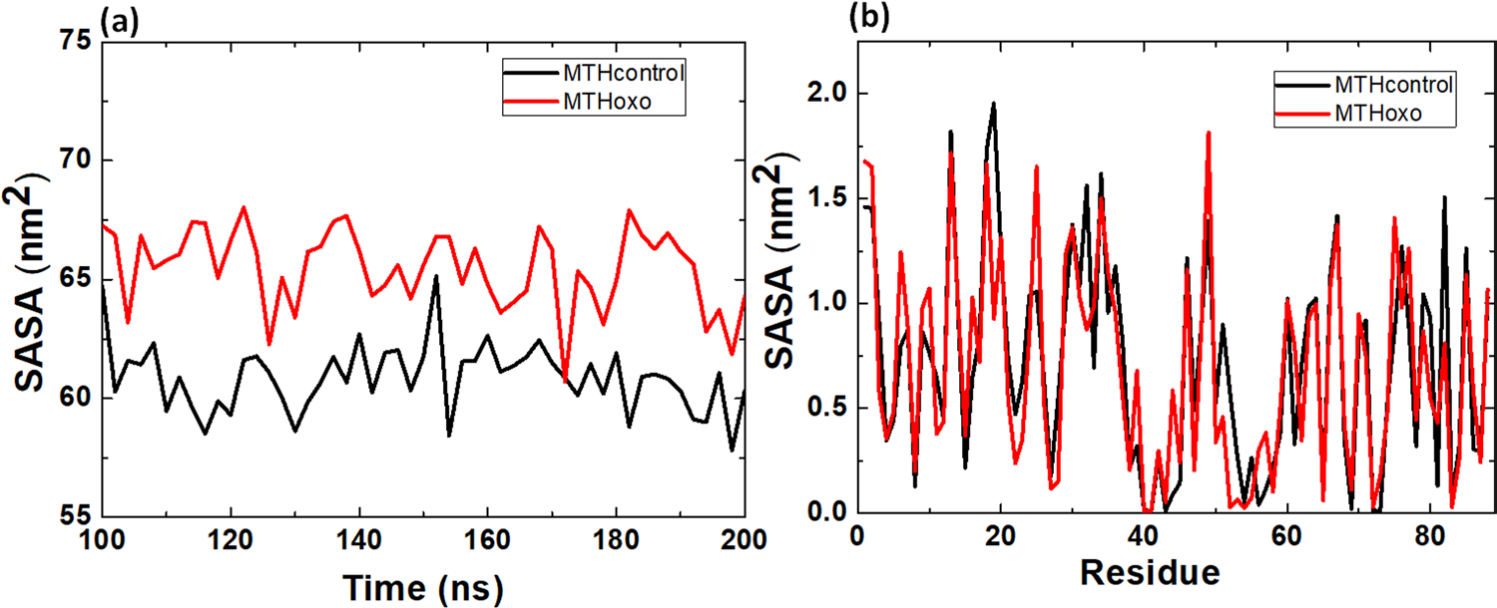
(**a**) SASA as function of time (ns) and (**b**) SASA per residue, for MTH control and MTHoxo.

Finally, we calculated the change in time of the solvent accessible surface area (SASA) for the native and oxidised MTH protein. We analysed the SASA values for the last 100 ns (Fig. 6a). The SASA provides information about the surface area of the protein that is accessible to the solvent. The average SASA values of MTH control and MTHoxo are 60.8 ± 1.4 and 65.4 ± 1.6 nm^2^, respectively. Moreover, we also calculated the SASA per residue for MTH control and MTHoxo. The SASA value is higher for MTHoxo as compared to MTH control for the residues between 3 and 11, and between 47 and 79 (Fig. 6b). The SASA calculation supports the ED and RMSD/RMSF results that after oxidation, the protein becomes more flexible, yielding a larger surface area of the protein that is accessible to the solvent.

These MD simulation results can be correlated with our experimental CD and fluorescence data, illustrating structural changes of MTH upon CAP treatment. Due to the structural distortion, the thermal and chemical denaturation of MTH increases upon CAP treatment.

## Conclusion

The purpose of this study was to determine the influence of ROS/RNS on the structural and thermodynamic deformation of protein from thermophilic bacteria, in order to obtain a better insight in the mechanisms for the inactivation of thermophilic bacteria upon CAP treatment. We used MTH as model protein from thermophilic bacteria, which we treated with DBD for 10, 15 and 20 mins. The CD, fluorescence and NMR results reveal that long enough CAP treatment (e.g. 20 min) can denature MTH to some extent. The thermal denaturation results show that 20 min of CAP treatment yields a drop in melting temperature by 5 °C, as well as a small change in chemical denaturation.

To better understand the experimental results, we have performed MD simulations for native MTH and oxidized MTH (which can be correlated to CAP treatment), and we observed that the RMSD, RMSF, ED and SASA change significantly for the oxidized MTH with respect to the native MTH. In general, we can conclude that the MTH structure can be destabilized/modified to some extent upon CAP treatment, but only at sufficiently high dose (i.e., long treatment time). This is a major achievement, because the protein from thermophilic bacteria are resistant to temperature and chemical denaturant, and therefore it is very difficult to destabilize them.

These findings shed new light on the effect of ROS/RNS on proteins from thermophilic bacteria, to better understand how ROS/RNS produced by CAP can inactivate thermophilic bacteria. The latter is of interest for the food industry, to protect food products, such as canned foods, juices, and milk products.

## Materials and Method Section

### Experimental Section

#### Cloning, purification of MTH1880 and Size exclusion chromatography

The MTH1880 gene was obtained from *Methanobacterium thermoautotropjicum* genomic DNA by PCR amplification and it was used as a template to clone MTH1880. Details of the purification are given in the supporting information and in previous work^56^. The size exclusion chromatography of the purified protein is given in Fig. S2.

#### Circular dichroism spectroscopy

CD spectroscopic studies were performed using J-815 spectrophotometry (Jasco, Japan) equipped with a Peltier system to control the temperature. The samples were pre-equilibrated at the desired temperature for 15 min, and the scan speed was fixed for adaptive sampling (error F 0.01) with a response time of 1 s with 1 nm bandwidth. The secondary MTH structures were monitored using a 1.0 mm path length cuvette. The concentration for the secondary MTH structure was 0.2 mg/ml, and each spectrum is taken as the average of six spectra. Each sample spectrum was obtained by subtracting the appropriate blank media without MTH from the experimental protein spectrum. The percentages of secondary structures were then calculated using Yang’s method^65^.

#### CD spectroscopy-based GdmCl studies

The stability studies were performed by temperature-controlled Jasco J-815 CD spectrometry. For each sample, the CD spectra were simultaneously measured from 200 to 250 nm at 25 °C. The ellipticity in the spectrum of native MTH in buffer was assumed to correspond to 100% folded protein, and the ellipticity in the spectrum of MTH with 6 M GdmCl was assumed to correspond to the unfolded protein. To denature the protein, MTH samples were dialyzed against buffer containing GdmCl for 24 h. After denaturation, GdmCl was removed from the MTH samples by extensive buffer exchange using dialysis for 24 h. Subsequently, the CD spectra were measured at 220 nm, to evaluate the change in conformation of MTH as a function of GdmCl concentration.

#### Temperature stability studies

Preliminary thermodynamic stability studies were performed by temperature-controlled J-815 spectrophotometry (Jasco, Japan) equipped with a Peltier system. For each sample, CD spectra were simultaneously measured at 220 nm as a function of temperature, from 25 to 100 °C. The sample was placed in a sealed cuvette to prevent water evaporation. The 220 nm ellipticity in the spectrum of native MTH in buffer at 25 °C was assumed to correspond to 100% folded protein, and the ellipticity at 100 °C was assumed to correspond to the unfolded protein. The folded fraction was computed as:

$${\rm{Fraction}}\,{\rm{Folded}}=\frac{{A}_{220}-{A}_{u}}{{A}_{f}-{A}_{u}}$$

In this formula, A_220_ is the absorbance between 25 and 100 °C, A_u_ is the absorbance of the unfolded protein at 100 °C, and A_f_ is the absorbance of the folded protein at 25 °C. In order to understand the change in protein conformation as a function of temperature, we studied the change in ellipticity at 220 nm.

#### Intrinsic fluorescence spectroscopy

Fluorescence spectra were measured using the LS55 spectrofluorophotometer (Perkin Elmer). The sample was contained in a 1 mL temperature-controlled cuvette (25 °C) and fluorescence spectra were acquired for wavelengths ranging from 270 to 500 nm. The excitation wavelength was fixed at 280 nm for the overall fluorescence emission. The slit widths for excitation and emission were both set at 10 nm. The MTH concentration was 0.5 mg/ml, and each spectrum was the average of six spectra.

#### Size exclusion chromatography

MTH samples (1 mg in 2 ml) were loaded onto a HiLoad™ 16/60 superdex™ 75 gel filtration column (GE Healthcare) equilibrated with sample buffer. The elution was carried out at a flow rate of 1.3 ml/min and monitored by absorbance at 280 nm. In all cases, the time that elapsed between separation and chromatography of the peaks was longer than 2 h.

#### NMR spectroscopy

NMR experiments were performed in a mixture of 90% water and 10% D_2_O NMR buffer, 10 mM HEPES (pH 7.0), 100 mM NaCl at 298 K on the Bruker DRX 500 MHz equipped with CryoProbe. Sequential resonance assignment was executed by ^1^H-^15^N HSQC^56^. For the NMR titration experiments, ^15^N labeled MTH was purified as in previous work^56^ and subsequently treated with plasma for 20 min. All collected spectra were processed and analyzed via XWIN NMR (Bruker Instruments, Karlsruhe, Germany), nmrPipe/nmrDraw (Biosym/Molecular simulation, Inc. San Diego, CA, USA) software, and the PINE-SPARKY program.

#### pH and temperature measurement

After plasma exposure to the buffer solution for different time durations, the pH and temperature of the buffer were measured using a pH meter (Eutech Instruments, Singapore) and Infrared (IR) camera (Fluke Ti100 Series Thermal Imaging Cameras, UK). All measurements were carried out in triplicate.

#### Dielectric barrier discharge (DBD) plasma

The DBD used for the experiments is explained in detail in previous work^38^. The V_rms_ is 1.23 kV and I_rms_ is 0.009 mA, and the discharge voltage is 0.9 kV. The on-time is 32 ms and the off-time is 154 ms, the electric energy per second is 0.623 J/sec. Optical emission spectra (OES) of the DBD emission were presented in our previous work^38^. They show weak emission lines for the molecular NO β, γ system between 200 and 250 nm, strong emission lines for the N_2_ second positive system between 300 and 420 nm, and a weak emission line of atomic oxygen at ≈777.5 nm.

#### Molecular dynamics simulations

**S**tructures of MTH were obtained from the (RCSB) protein data bank website (http://www.rcsb.org; the PDB ID was 1IQS)^56^. The MD simulations were performed with the GROMACS 5.1 package^66^ using the GROMOS54A7 force field^67^. The protein was solvated with water, described by the SPC explicit solvent model^68^. The protein was placed in a cubic box of volume 299.59 nm^3^. To perform simulations for a neutral system, Na^+^ or Cl^−^ ions were added to the system by randomly replacing the water molecules in the simulation box. The system energy was minimized with the steepest descent method for 50000 timesteps, and 1000 kJ/mol/nm energy tolerance for convergence of the minimization process. Furthermore, the system was equilibrated at 300 K in the NPT ensemble for 200 ns. Subsequently, the protein position restraint was removed and MD simulations were performed for 200 ns at a temperature of 300 K and pressure of 1 bar using the leap-frog algorithm to numerically integrate the equations of motion with a time step of 2 fs. Finally, we calculated the RMSD, RMSF, ED and SASA values to check the stability, flexibility and surface area of the protein that is accessible to the solvent. Besides simulating the native MTH structure, we also performed the same simulations to an oxidised form of MTH, to better understand the effect of CAP treatment (oxidation) on the MTH stability. The structure of oxidised MTH1880 was based on the NMR spectra, i.e., assuming oxidised His52 and Tyr59. More specifically, they were oxidised into 2-oxo-histidine and 3,4-dihydroxyphenylalanine, respectively, which are the most probable oxidised structures after plasma treatment^64^. For oxidised MTH, the GROMACS force field parameters were obtained from reference^69,70^.

## Electronic supplementary material

Supporting Information
