# Supplementary material for: CAP modifies the structure of a model protein from thermophilic bacteria: mechanisms of CAP-mediated inactivation
Source: Sci Rep. 2018 Jul 5;8:10218. doi: 10.1038/s41598-018-28600-w (PMC6033864; doi:10.1038/s41598-018-28600-w)
Supplement: Supplementary file 1 — Supporting Information [file 41598_2018_28600_MOESM1_ESM.docx]

**Supporting Information**

**CAP modifies the structure of a model protein from thermophilic bacteria: mechanisms of CAP-mediated inactivation**

Pankaj Attri^1^, Jeongmin Han^2^, Sooho Choi^2^, Eun Ha Choi^3^, Annemie Bogaerts*^1^, Weontae Lee*^2^

^1^Research Group PLASMANT, Department of Chemistry, University of Antwerp, Universiteitsplein 1, B-2610 Antwerp, Belgium

^2^Department of Biochemistry, College of Life Science & Biotechnology, Yonsei University, 134 Shinchon-Dong, Seodaemoon-Gu, Seoul, 120-749, Korea.

^3^Department of Electrical and Biological Physics, Kwangwoon University, Seoul 01897, Korea

**Cloning, purification of MTH1880 and Size exclusion chromatography**. The MTH1880 gene was obtained from Methanobacterium thermoautotropjicum genomic DNA by PCR amplification and it was used as a template to clone MTH1880. Each gene was cloned using BamHI and XhoI sites in the vector pET21b (Novagen), as a fusion protein with an N-terminal hexahistidine affinity tag and TEV cleavage site (ENLYFQG). Escherichia coli (Strain BL21 DE3) was the host for non-labeled MTH1880 or 15N labeled MTH1880 and were grown in LB and M9 minimal media containing (U-15N) at 37°C. Subsequently, 1 mM IPTG was added to induce expression for 15 h at 25°C. The cells were harvested by centrifugation and stored at -80°C. The harvested cells were disrupted by sonication in lysis buffer (25 mM sodium phosphate, 300 mM NaCl, and protease inhibitor cocktail (Roche), pH 8.0). The (His)6 tag fusion proteins were purified with immobilized metal affinity chromatography on a Ni-NTA column (Amersham Pharmacia) and cleaved by tobacco etch virus protease for 15 h. The purified protein was concentrated using Amicon Ultra centrifugal filter devices (Millipore, cut off 5 kDa).

The MTH1880 samples (1 mg in 2 ml, pre-incubated) were loaded onto a HiLoad™ 16/60 superdex™75 gel filtration column (GE Healthcare) equilibrated with sample buffer. The elution was carried out at a flow rate of 1.3 ml/min and monitored by absorbance at 280 nm. In all cases, the time that elapsed between separation and chromatography of the peaks was longer than 2 h.


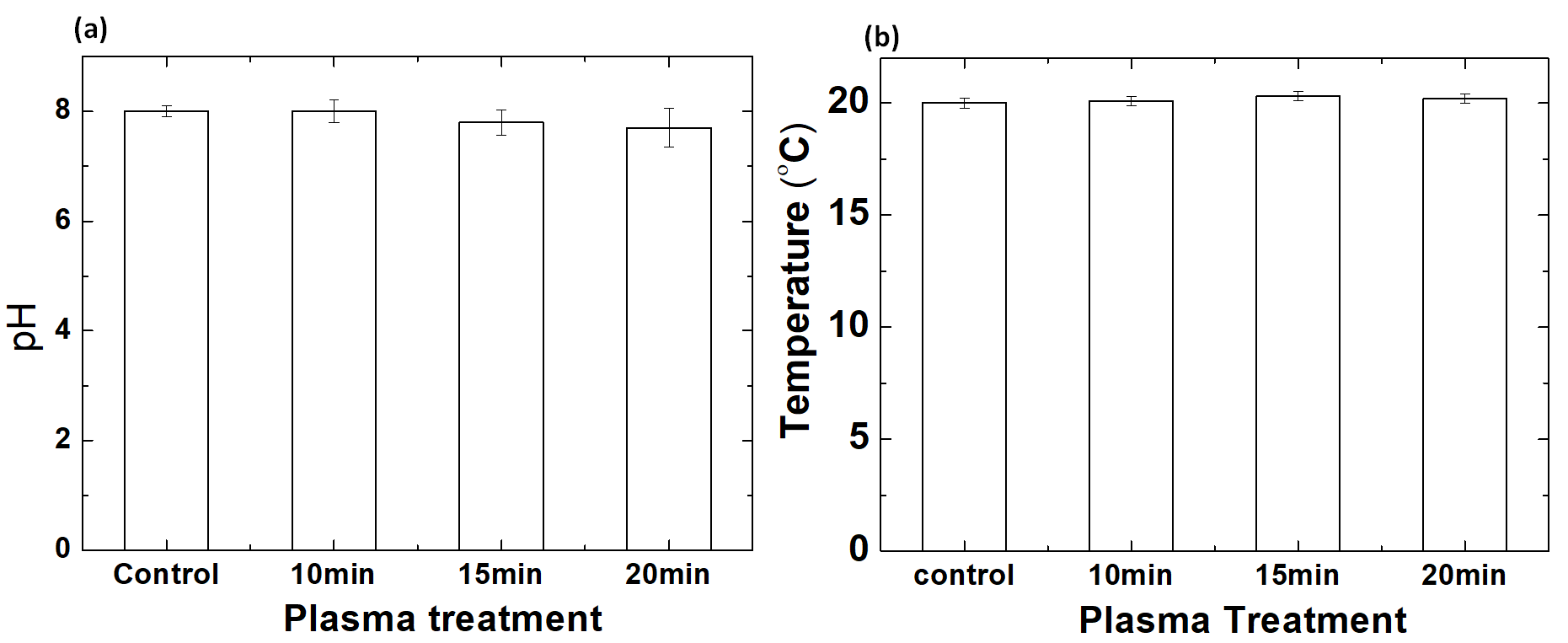


**Figure S1:** (a) Change in pH and (b) change in temperature of the buffer solution after plasma treatment for different times.


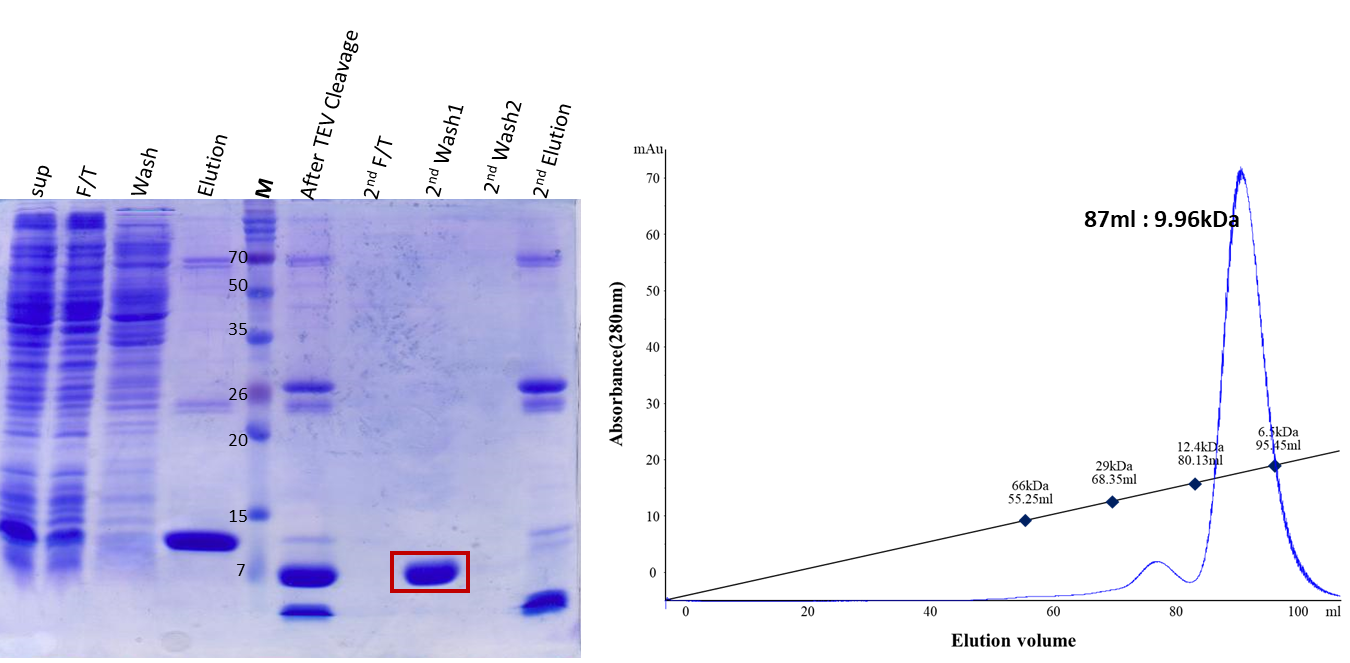


**Figure S2**: size exclusion chromatography of the purified protein MTH1880.
